# Prescriptions

**Published:** 1897-05

**Authors:** 


#### GONORRHŒA.

℞ Potassii citratis........ ℥ ss—j

Spts. limonis........... ʒ ss.

Syr. simplicis.......... ℥ ij.

Aquæ.................. ℥ j.

M. Sig.: A dessertspoonful, well diluted, three or four times daily, fasting. (*In first stage.*)

*Van Buren and Keyes.*

#### FROSTBITE.

℞ Tinct. benzoini......... ʒ ij.

Olei lini.................. ʒ iv

Ceræ flav.................. ʒ ij.

Glycerine................ q. s.

Ft.ungt.

M. Apply locally.

*Reveil.*

#### STOMATITIS.

℞ Borax.................. 45 grs
Salicylate of sodium....75 grs
Tincture of myrrh......1 dr.
Syrup and water, of each, ½ oz.
M. Sig.: Mouth wash.

#### BBONCHITIS.

℞. Vini ipecac............... ʒ j

Tinct. scillæ............. ʒ ij.

Syr. tolutan............... ʒ v.

Aquæ...................... ℥ j.

M. Sig.: A teaspoonful every three or four hours.

#### HEMORRHOIDS.

℞. Acidi carbolici.............. ʒij.

Acidi tannici ............. ʒj.

Alcoholis................. ʒiv.

Glycerinæ.................. ℥j.

M. Sig.: Hypodermic injection for piles.—*Girard.*

